# Comparison of migration behavior between single and dual lag screw implants for intertrochanteric fracture fixation

**DOI:** 10.1186/1749-799X-4-16

**Published:** 2009-05-18

**Authors:** George K Kouvidis, Mark B Sommers, Peter V Giannoudis, Pavlos G Katonis, Michael Bottlang

**Affiliations:** 1 Department of Orthopaedic Surgery and Traumatology, University Of Crete, Heraklion, Greece; 2 Biomechanics Laboratory, Legacy Research & Technology Center, Portland, Oregon 97215, USA; 3 Trauma & Orthopaedic Surgery School Of Medicine, University of Leeds, Leeds General Infirmary, Great George Street, Leeds, LS1 3EX, UK

## Abstract

**Background:**

Lag screw cut-out failure following fixation of unstable intertrochanteric fractures in osteoporotic bone remains an unsolved challenge. This study tested if resistance to cut-out failure can be improved by using a dual lag screw implant in place of a single lag screw implant. Migration behavior and cut-out resistance of a single and a dual lag screw implant were comparatively evaluated in surrogate specimens using an established laboratory model of hip screw cut-out failure.

**Methods:**

Five dual lag screw implants (Endovis, Citieffe) and five single lag screw implants (DHS, Synthes) were tested in the Hip Implant Performance Simulator (HIPS) of the Legacy Biomechanics Laboratory. This model simulated osteoporotic bone, an unstable fracture, and biaxial rocking motion representative of hip loading during normal gait. All constructs were loaded up to 20,000 cycles of 1.45 kN peak magnitude under biaxial rocking motion. The migration kinematics was continuously monitored with 6-degrees of freedom motion tracking system and the number of cycles to implant cut-out was recorded.

**Results:**

The dual lag screw implant exhibited significantly less migration and sustained more loading cycles in comparison to the DHS single lag screw. All DHS constructs failed before 20,000 cycles, on average at 6,638 ± 2,837 cycles either by cut-out or permanent screw bending. At failure, DHS constructs exhibited 10.8 ± 2.3° varus collapse and 15.5 ± 9.5° rotation around the lag screw axis. Four out of five dual screws constructs sustained 20,000 loading cycles. One dual screw specimens sustained cut-out by medial migration of the distal screw after 10,054 cycles. At test end, varus collapse and neck rotation in dual screws implants advanced to 3.7 ± 1.7° and 1.6 ± 1.0°, respectively.

**Conclusion:**

The single and double lag screw implants demonstrated a significantly different migration resistance in surrogate specimens under gait loading simulation with the HIPS model. In this model, the double screw construct provided significantly greater resistance against varus collapse and neck rotation in comparison to a standard DHS lag screw implant.

## Introduction

Operative treatment for hip fractures was introduced in the 1950s with the expectation of improved functional outcome and a reduction of the complications associated with immobilisation and prolonged bed rest [1-3].

Since then a variety of different implants has been used either extramedullary or intramedullary in nature. The most commonly used extramedullary implant is the sliding hip screw (SHS) with side plate. It is currently considered the gold standard for fixation of extracapsular hip fractures as well as the implant that any new design should be compared with [4-6].

The SHS has been shown to produce good results; however, complications are frequent, particularly in unstable fractures [7-9].

Post-operative implant-related complications have been reported in a recent meta-analysis of 16 studies. Risks ranged from 0 to 23% (median 6%) in patients treated with intramedullary devices and from 0 to 7% (median 3%) in patients treated with SHS devices [10]. The most common cause of failure is reported to be varus collapse and cutting-out of the lag screw through the femoral head [11,12].

Intramedullary (IM) implants have been associated with an increased risk of intraoperative and postoperative femur fractures compared with sliding hip screws [13-16]. This increased fracture incidence has been linked to stress concentration at the tip of the IM nail, stress concentration at the distal locking bolt, and reaming of the proximal femur to accommodate the increased proximal diameter of the nail necessary to allow a large diameter lag screw to pass through the nail [13,17-19].

Recently, IM nails have been introduced that employ two small-diameter lag screws that enable a smaller diameter of the proximal nail segment [20,21]. The decreased proximal nail diameter requires less, or even no reaming of the proximal femur and potentially lowers the incidence of iatrogenic proximal femur fracture. Furthermore, two proximal screws theoretically provide greater rotational control of the femoral head fragment than a single screw [20,22]. There are concerns, however, that the smaller diameter screws would be more prone to migration through the femoral head and increase the incidence of screw cut-out [20].

In a biomechanical comparison by Erik N Kubiak et al. between a dual, trochanteric antegrade nail (TAN) and a single lag screw implant (intramedullary hip screw IMHS), the two constructs showed equivalent rigidity and stability in all parameters. The dual screw implant had a significantly greater ultimate failure load [23]. Mickael Ropars et al. in a recent study, compared two minimally invasive implants; one with dual, and one with single cephalic lag screws [24]. They concluded that both implants have biomechanical properties which are as favourable as conventional hip screws, and that loading and mode of failure were found to be similar. In both studies and for static and cyclic loading the specimens were only loaded in the vertical direction. They did not accounted for the multi-planar loading seen by the hip during level walking. However it is well known that the implant-bone interface is subjected to combine axial and torsion loading during walking that seems to significantly affect the lag screw migration [25].

Larry Ehmke et al. developed a hip implant performance simulator (HIPS) that can reproduce the dynamic multi-planar hip forces seen during level walking. Their HIPS system evaluates lag screw migration in a pertrochanteric fracture model under more physiologic loading conditions. They chose the biaxial rocking motion (BRM) technology to simulate multi-planar forces with a loading protocol accounting for hip flexion-extension, ab-adduction and a double peak load history. The so-called BRM design is the most commonly used wear test device for prosthetic hip joints [25-28].

To our knowledge, fixation strength of double-screw hip implants has not been tested in a laboratory cut-out simulator under dynamic multi-plantar loading to determine if an additional screw does provide significantly improved migration resistance as compared to a standard single screw implant. This study therefore investigated the migration behaviour and cut-out resistance of a novel double lag screw implant in comparison to a commonly used single lag screw implant under physiologic multi-planar loading in an established laboratory model [29,30].

## Methods

### Implants

**Figure 1:**
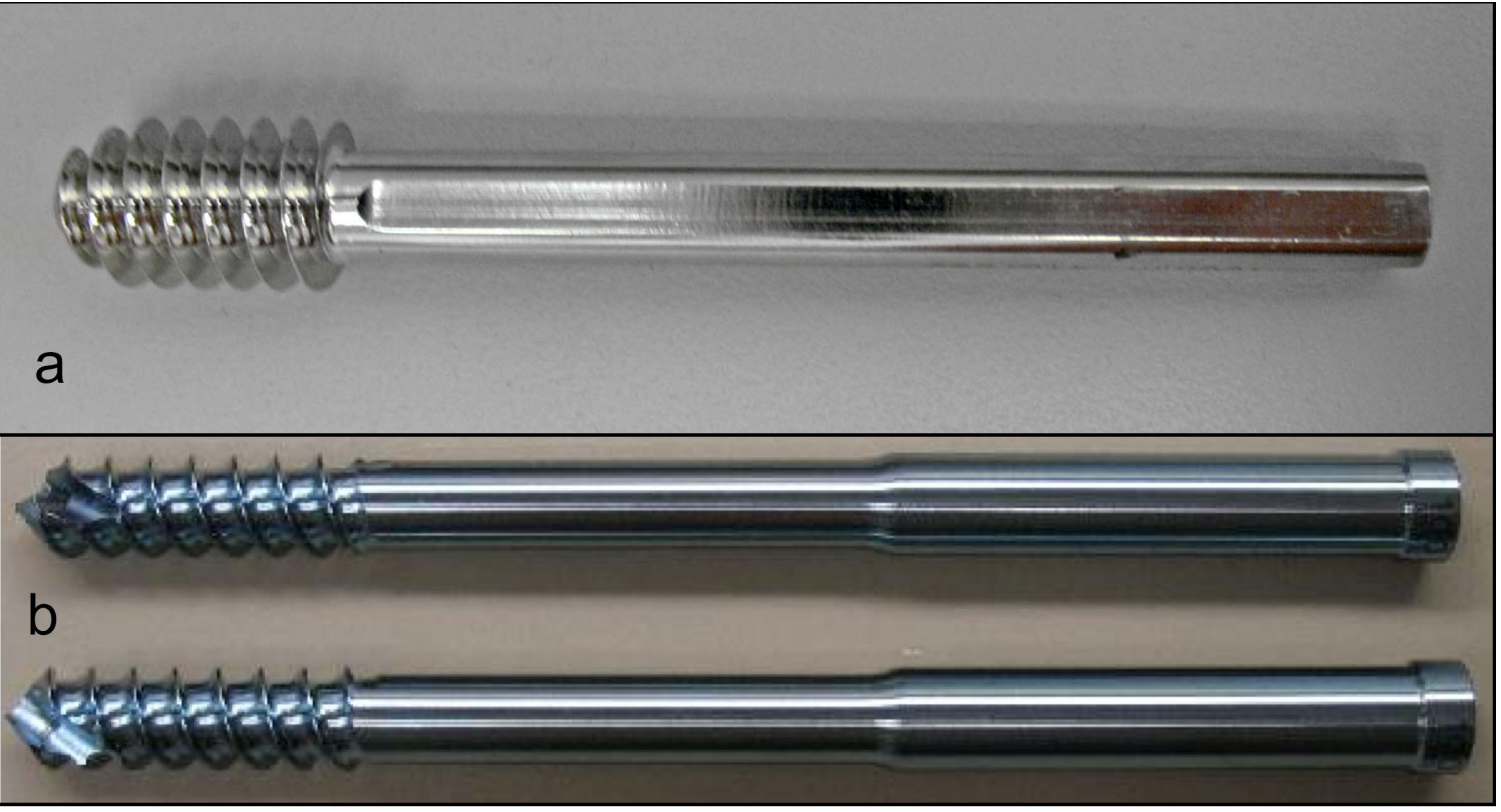
**a) DHS single lag screw, and b) Endovis dual lag screws with self-drilling and self-tapping screw tip**.

As the gold-standard for single lag screw implants, five dynamic hip screws (DHS, Synthes, West Chester, PA) made of stainless steel were tested. The DHS lag screws had a shaft diameter of 7.8 mm and an outer thread diameter of 12.5 mm (Figure 1a). To investigate if a dual lag screw implant can provide greater migration resistance, five pairs of Cephalic Screws of a cephalomedullary device (Endovis, Citieffe, Italy) made of titanium were tested. The lag screws had a shaft diameter of 7.5 mm and an outer thread diameter of 9.7 mm. They had a self-drilling and self-tapping screw tip design (Figure 1b).

### Surrogate Specimens

Lag screw fixation was tested in surrogate femoral head and neck specimens of 50 mm diameter machined from cellular polyurethane foam (#1522-11, Pacific Research Inc., Vashon, Washington, USA). These specimens had a density of 12.5pcf (0.2 g/cm^3^) with 4 MPa compressive strength and 48 MPa elasticity modulus (E-modulus) to simulate mildly osteoporotic bone, as validated in a previous study [30]. These material properties correspond to the osteoporotic range of human cancellous bone, with 2–21 MPa compressive strength and 5–104 MPa E-modulus [31,32]. Surrogate specimens were used as a cancellous bone substitute to maximize result reproducibility [29,30]. The surrogate specimens were placed in a 6 mm thick, polished steel shell to provide a rigid, spherical interface for delivery of dynamic loading.

### Implant Insertion

**Figure 2:**
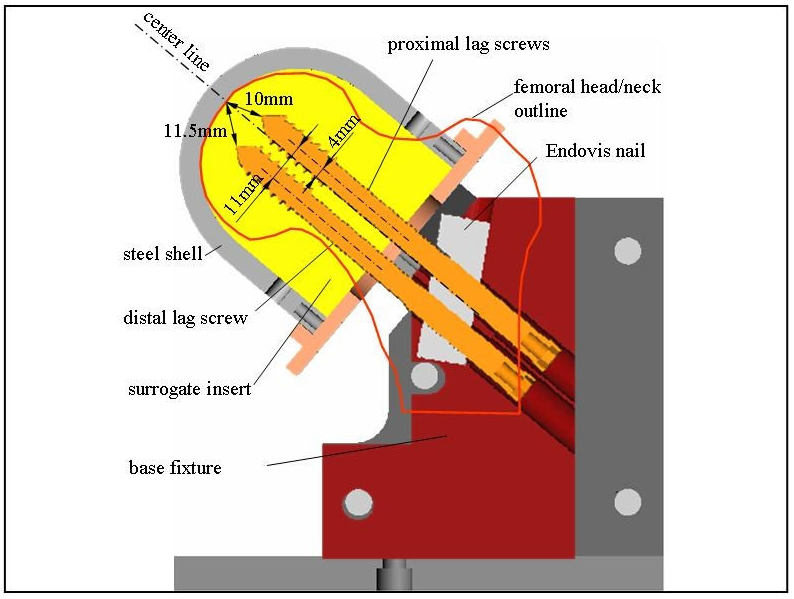
**Positioning of Endovis cephalic screws in the femoral head/neck surrogate, shown in association with proximal nail segment and base fixture**.

All implants were inserted according to the manufacturer's guidelines. For DHS lag screws, surrogate specimens were reamed but not tapped. The lag screw was placed centrally within the femoral head surrogate and advanced to a depth leaving 20 mm tip-to-apex distance (TAD) [33]. This corresponds to a 10 mm distance of the screw tip to the femoral head apex in both the antero-posterior and lateral radiographic view. The two cephalic screws were inserted without pre-drilling, reaming or tapping. Both screws were inserted to the same depth, yielding a TAD distance of the superior screw of 20 mm (Figure 2). Accurate screw insertion was supported by a custom-made insertion guide, to ensure proper distance of the screws to each other and proper location within the femoral head surrogate.

### Experimental Setup

**Figure 3:**
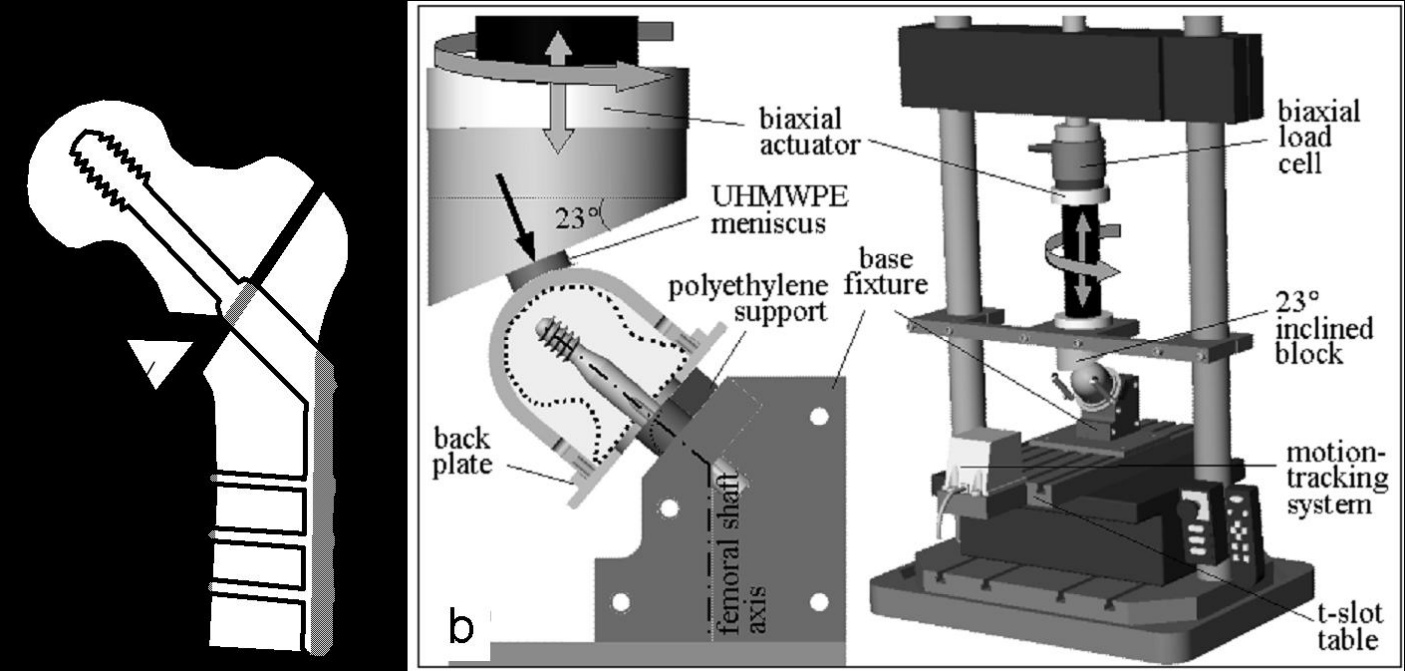
**HIPS model for testing of lag screw fixation strength**: a) unstable fracture model; and b) HIPS base fixture, shown in cross-sectional view and in assembly with material test system for application of biaxial rocking motion representative of hip loading during level walking.

Specimens were transferred for testing in the HIPS of the Legacy Biomechanics Laboratory [29]. This model has been validated for simulation of lag screw migration and cut-out in a clinically relevant worst-case scenario, accounting for osteoporotic bone, an unstable intertrochanteric fracture (OTA classification 31-A.2), and gait-cycle loading (Figure 3a). The base fixture of the HIPS system modeled a femoral shaft with its anatomic axis aligned perpendicular to the horizontal plane. The proximal aspect of the base fixture was designed to simulate a pertrochanteric fracture line oriented 40° to the anatomic axis of the femoral shaft (Figure 3b). The back plate of the femoral head steel shell had a 40 mm diameter hole to ensure unconstrained shear translation of the lag screw shaft in the femoral neck. This back plate rested against a polyethylene support attached to the base plate, reproducing the constraints characteristic of a reduced, but unstable pertrochanteric fracture. Specifically, this support simulated abutment of the fracture surfaces after completion of lag screw sliding, while still allowing femoral head varus collapse and rotation, as in the case of an unstable fracture with deficient posteromedial neck support. To replicate clinically relevant sliding conditions, the clamping part in the base plate incorporated either, the barrel and side-plate, in case of the DHS, or the section of the cephalomedullary nail with the two support holes for the lag screws.

### Loading

BRM, representative for level walking, was produced using concurrent axial loading and rotational displacement controlled by a biaxial material test system (Instron 8874, Canton, Massachusetts, USA). A dynamic, double-peak loading regimen of 1.45 kN peak load approximating two times bodyweight was applied at 1 Hz to the steel shell over a polyethylene meniscus. The meniscus traced a path on the femoral head similar to the path of resultant force vectors during level walking. Concurrent flexion-extension and abduction-adduction motion were superimposed by sinusoidal rotation of a 23° inclined block affixed to the actuator (Figure 3b). This 23° incline accounted for an 18° resultant joint load vector, plus 5° of valgus of the femoral shaft axis. Exaggerated walking kinematics of the left limb was simulated by ± 75° rotation of the actuator, which resulted in a 45° arc of flexion-extension and a 17° arc of ab-adduction. Implants were exercised either until failure or up to 20,000 load cycles, whichever occurred first.

### Outcome Measures

Three dependent outcome variables were reported, one of which describes the cut-out resistance (N_F_), while the remaining two (α_Neck_, α_Varus_) describe the migration kinematics. The number of load cycles to implant failure, N_F_, was registered by the material test system. Cut-out failure was detected by means of electrical conductivity between the implant and the steel shell, which triggered an instantaneous stop of the test system to preserve the cut-out stage. Femoral head migration kinematics was analyzed in terms of varus collapse (α_varus_) and rotation around the neck axis (α_neck_). These migration kinematics data were continuously recorded with an electromagnetic motion tracking system (PcBird, Ascension Tech., Burlington, Vermont, USA). To suppress distortion of motion tracking data by ferromagnetic interference, a non-ferrous experimental platform and actuator extension was implemented. Additionally for dual screws implants, the migration of lag screws in axial direction was assessed by measuring their position with a digital caliper before and after testing.

### Statistical Analysis

Differences in migration α_Neck _and α_Varus _between implants were tested at discrete time points during the loading history at a confidence level of α = 0.05 using two-tailed Student's t-tests for unpaired samples.

## Results

### Lag Screw Cut-Out

**Figure 4:**
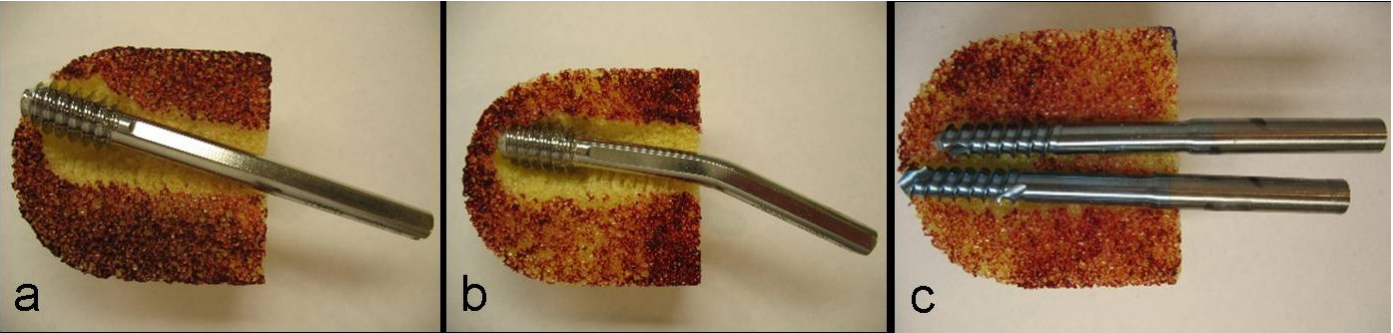
**Lag screw failure modes**: a) DHS varus collapse and subsequent cut-out failure, b) lag screw bending in absence of cut-out, and c) axial migration of distal Endovis screw, leading to cut-out despite minimal varus collapse.

All DHS implants failed before 20,000 loading cycles, on average after 6,638 ± 2,837 cycles. Two out of five DHS specimens failed due to implant cut-out after 11,161 and 4,486 cycles (Figure 4a). The remaining three DHS implants exhibited lag screw bending in absence of cut-out failure after on average 5,848 ± 1,616 cycles (Figure 4b). Four out of five dual screws implants sustained 20,000 loading cycles. Only one dual screws implant exhibited cut-out by medial migration of the inferior screw, which occurred after 10,054 load cycles. No bending was observed in dual lag screws (Figure 4c).

### Migration

At failure, DHS implants migrated on average to α_varus _= 10.8° ± 2.3° and α_neck _= 15.5° ± 9.5°. Migration in dual Cephalic screws advanced to α_varus _= 3.7° ± 1.7° and α_neck _= 1.6° ± 1.0° after completion of 20,000 cycles or at failure. In addition, axial migration of dual screws was observed. The proximal and distal screws migrated medially by on average 0.3 ± 0.8 mm and 4.9 ± 3.0 mm medially, respectively.

**Figure 5:**
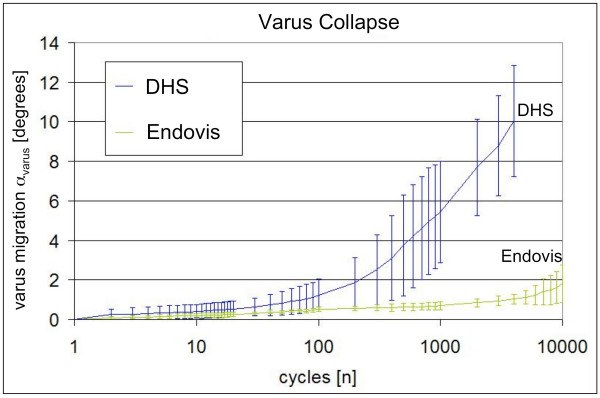
**Progression of varus collapse under dynamic loading for single (DHS) and double (Endovis) lag screw constructs**.

**Figure 6:**
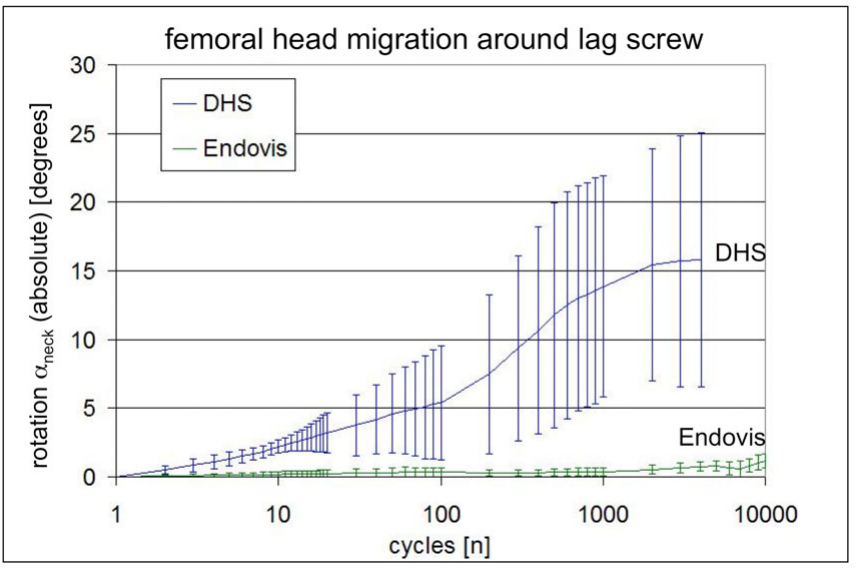
**Progression of femoral head rotation around the lag screw under dynamic loading for single (DHS) and double (Endovis) lag screw constructs**.

Histories of the average migration were calculated for each implant, specific for α_varus _(Figure 5) and to α_neck _(Figure 6). Endpoints of the average migration histories represent the last average data point, collected at the earliest failure among the individual tests. The double screw construct was more stable than the DHS in both α_varus _and α_neck_. Dual screws implants demonstrated consistently less varus collapse, which was significantly below that of DHS implants at and after 300 loading cycles. Average neck rotation histories were based on absolute neck rotation, since the direction of neck rotation appeared to be random. After the third loading cycle, neck rotation was significantly lower in Endovis implants as compared to DHS implants.

## Discussion

There are previous published biomechanical studies [23,24] that directly compared the stability of single and dual lag screw implants used for treatment of intertrochanteric hip fractures. The authors of these prior studies concluded that the two constructs showed equivalent rigidity and stability, that biomechanical properties were as favourable as conventional hip screws, and that the dual lag screw implants had a greater ultimate failure load. However their loading parameters in these prior studies did not reflect the physiological forces that act on the hip during level walking since loading was applied to the specimens uni-axially in the coronal plane. Coronal plane loading represents the forces across the hip in single leg stance of the gait cycle [34]. The HIPS model was specifically designed to simulate loading vectors experienced by the proximal femur during ambulation [29]. It employs BRM, a well-validated protocol for producing hip motion using a dynamic, multi-planar double peak loading regimen. Ehmke et al. demonstrated that, cut-out mechanisms differed between multi-planar BRM loading and uni-axial loading. Only the BRM loading model resulted in cut-out that occurred by combined varus collapse and neck rotation. Moreover, they found that the initial motion was rotation about the lag screw, followed by varus collapse [29].

The DHS lag screws were placed in near perfect position, according to Baumgaertner et al. [33] with a tip-to-apex distance of 20 mm to 32 mm, depending on whether the steel shell is considered part of the articular layer, or part of the femoral head, respectively. According to the same principles, the proximal Endovis screw was placed closer to the central axis of the femoral head leaving space in the distal third of the femoral head and neck for the distal screw. The tip-to-apex distance was 20 mm for the proximal and 23 mm for the distal screw. This relatively eccentric placement of the Endovis screws preserves bone stock in the upper part of the femoral head which may further improve cut-out resistance.

Both implants were inserted into the surrogate specimens according to the manufacturer's instructions using the suggested instrumentation in the same manner as in real surgery. Differences in insertion methods between the two implants are due to differences in design of the implant's tip. The two cephalic screws had a self drilling and shelf taping design. It is known that insertion torque and pull-out forces for these screws are similar to pre-tapped screws [35]. Moreover there are no data from the literature to support that such differences in insertion methods may have influenced the cut-out resistance of our screws in order to affect our results. In a recent biomechanical comparison of conventional DHS and DHS Blade the authors noticed an enhanced cut out resistance of the DHS Blade. The main difference between the two implants was the implantation technique, pre-drilling for the DHS and impaction for the blade. They suggested that the underlying mechanism for improved purchase of the blade implant is unclear, but bone-compaction is deemed to play a major role. However they finally concluded that maximizing the bone content around the implant forgoing pre-drilling does not necessarily enhance the cut-out resistance, since mainly elastic deformation seems to contribute to the implant anchorage. The importance of the implantation technique with or without pre-drilling is therefore decreased [36].

In addition to differences in insertion between screws, there are some small differences in the geometry of the screws as well. It is accepted that implant development and design remains a major approach in the efforts for developing superior treatment concepts for osteoporotic proximal femur fractures [37]. A plethora of published biomechanical studies have attempted to define the optimum shape and size of the ideal implant for these challenging fractures. It is obvious that this ideal implant is still unknown up to date since cut-out remains one of the major clinical challenges in the field of osteoporotic proximal femur fractures [38]. With these thoughts in mind the small differences in shaft diameter, and outer thread diameter of our implants, in relation to fixation strength and migration, is extremely difficult to investigate. The only parameter, in our model that can explain the superior biomechanical properties of dual cephalic screws seems to be the presence of the second screw, which can better control torsional forces.

Clinical studies have consistently failed to find significant differences between implant designs with regard to lag screw cut-out [11,39,40]. The clinical incidence of implant-related cut-out is masked by the high variability in bone quality, fracture pattern, quality of reduction, and implant placement. Using surrogate specimens in a controlled laboratory model provided sufficient reproducibility to enable detection of significant differences in migration resistance between the two implants tested. Similarity in migration kinematics and cut-out failure modes between cadaveric and surrogate specimens tested in the HIPS simulator has been demonstrated previously [29], and further supports the relevance of the present findings obtained within surrogate specimens.

The fixation strength of the dual lag screw construct was found to be significantly greater than the classic DHS when multidirectional dynamic forces were used for loading. In their biomechanical study, Kubiak et al. found that TAN, a dual lag screw intramedullary implant, provided significantly stronger fixation than the IMHS when loaded to failure in an unstable intertrochanteric hip fracture model [23]. These findings support Ingman's contention that the increased rotational control of the femoral head afforded by two screws would decrease femoral head cut-out [21]. In the present study, the double screws constructs demonstrated significantly less rotation (1.2°) than the DHS constructs (15.8°). Assuming that rotation about the femoral neck contributes to a loss of reduction and fixation stability, the superior rotational stability of dual screws implants may therefore be in part responsible for its increased resistance to varus migration and cut-out failure.

All DHS implants failed before 20,000 loading cycles, on average after 6,638 ± 2,837 cycles. Three out of five DHS specimens experienced implant bending before producing cut-out. Ehmke et al. utilized the HIPS system and found that all implants tested survived 20,000 cycles in bone surrogates. A basic difference between the present study and that of Ehmke's was the mechanical characteristics of the implants each study tested. Ehmke et al. tested a gamma nail lag screw with 12 mm shaft and 12 mm outer thread diameter in contrast to our study that tested the classic DHS lag screw with only 7.8 mm shaft and 12.5 mm outer thread diameter. Implant bending of DHS screws has been very rarely reported in clinical studies. However, in biomechanical studies it has been reported that the failure mode associated with the DHS lag screw in hard bone was screw bending rather than cut-out [8]. Bending is highly unusual and rarely if ever seen in osteoporotic patients and someone may say that our model is questionable at best. It is true that the surrogate foam chosen for our test proved to be harder that we expected. Moreover in biomechanical studies the high load levels such as 1.45 kN peak, or two times body weight, were chosen to reliably induce onset of implant migration within a clinically realistic number of loading cycles for each of the implants tested. When loading remained below a certain threshold, initiation of implant migration did not occur for a prolonged amount of time, during which fracture healing can occur clinically. Implant bending is therefore very rare in clinical practice but not so unusual in biomechanical studies.

However the critical point of our study is the amount of migration measured that is directly related to the fixation strength of the implants. Even in these "mild osteoporotic" surrogates dual screw implants shows superior stability compared to the classic DHS implants, and the measurements were comparable and reproducible.

An important result of dual lag screws is the substantial amount of axial medial migration of the inferior screw that was noted after 10,054 load cycles. The axial migration of lag screws has been described as the "Z effect" phenomenon [41]. This is a rarely reported mechanism of implant failure of the femoral neck element and has been described primarily for two-screw devices such as the proximal femoral nail (PFN, Synthes, Switzerland) [41]. The incidence of this phenomenon remains unknown and the biomechanical explanation for the medial migration of the femoral neck element has not been elucidated in the literature [42].

In our model both screws migrated medially by on average 0.3 ± 0.8 mm for the superior screw and substantially more (4.9 ± 3.0 mm) for the inferior screw. However only one inferior screw exhibited cut-out by medial axial migration, which occurred after 10,054 load cycles. To prevent the "Z effect" in clinical practice, Jinn Lin [43] emphasized inserting the inferior lag screw as close as possible to – or even right on – the inferior cortex of the femoral neck. Doing so could prevent this phenomenon and could also increase the bone mass to resist screw cut-out.

Limitations to the HIPS system must be recognized. Our neck constraint assumed that the fracture had undergone maximum collapse, but had not begun migration of the lag screw. This assumption has precedence in a study by Friedl and Clausen, which used neck constraints similar to those used in the HIPS model to simulate OTA 31-A3 pertrochanteric fractures [44]. Additionally, we did not load all specimens to failure, but ceased loading at 20,000 cycles. Our previous studies have clearly shown that the onset and pathway of migration is a more sensitive tool to determine fixation strength than cut-out [29,30]. As a further limitation, results of our study only describe implant performance in regard to cut out failure in absence of fracture healing and do not take into account alternative failure modes. A prospective randomized clinical trial would be required in order to determine if these favorable biomechanical results of double screw implants can be reproduced in clinical practice.

In conclusion, double screw construct provided significantly greater resistance against varus collapse and neck rotation in comparison to the gold-standard DHS implant when tested in the HIPS model under conditions representative of an unstable fracture and mild osteoporotic bone.

## Competing interests

Financial support was provided by a grant from the Plus Orthopaedics Hellas S.A.

## Authors' contributions

GK, PG and MB have contributed to the conception/design, data interpretation, and drafting/revising of the manuscript. MS has contributed to perform the routine aspects of the study including, biomechanical tests, data capture and statistical analysis. PK has been involved in revising critically the manuscript. All authors approved the final manuscript.
